# Trdmt1 3'-untranslated region functions as a competing endogenous RNA in leukemia HL-60 cell differentiation

**DOI:** 10.1590/1414-431X20209869

**Published:** 2020-12-09

**Authors:** Sha Xu, Jun Xiong, Minjuan Wu, Yu Yang, Junfeng Jiang, Haitao Ni, Yunpeng Zhao, Yue Wang

**Affiliations:** 1 Institute of Translational Medicine, Navy Medical University, Shanghai, China; 2 Department of Embryology and Histology, Navy Medical University, Shanghai, China

**Keywords:** Acute myeloid leukemia, Trdmt1, ceRNA, miRNA-181a, C/EBPα

## Abstract

Severe blockage in myeloid differentiation is the hallmark of acute myeloid leukemia (AML). Trdmt1 plays an important role in hematopoiesis. However, little is known about the function of Trdmt1 in AML cell differentiation. In the present study, Trdmt1 was up-regulated and miR-181a was down-regulated significantly during human leukemia HL-60 cell differentiation after TAT-CT3 fusion protein treatment. Accordingly, miR-181a overexpression in HL-60 cells inhibited granulocytic maturation. In addition, our “rescue” assay demonstrated that Trdmt1 3′-untranslated region promoted myeloid differentiation of HL-60 cells by sequestering miR-181a and up-regulating C/EBPα (a critical factor for normal myelopoiesis) via its competing endogenous RNA (ceRNA) activity on miR-181a. These findings revealed an unrecognized role of Trdmt1 as a potential ceRNA for therapeutic targets in AML.

## Introduction

Acute myeloid leukemia (AML) is a myeloproliferative disorder characterized by maturation arrest within the myeloid lineage. Multiple factors including genomic mutations, epigenetic changes, or gene expression disorders contribute to the development of AML. Studies have revealed that blockage in myeloid differentiation is the hallmark of AML (1). Therefore, finding the key regulators that promote human AML cell differentiation may be the potential approach to the treatment of AML.

MicroRNAs (miRNAs) regulate gene expression by pairing with miRNA response elements (MREs) generally located in the 3′ untranslated region (UTR) of target mRNAs and participate in the development of diseases including AML (2). In recent years, some studies have reported a type of miRNA underlying post-transcriptional regulation, known as competitive endogenous RNA (ceRNA). The ceRNA hypothesis proposes that protein-coding messenger RNA and non-coding RNA transcripts compete for binding to common miRNAs through cross-talking with and co-regulating each other by using MREs (3). Pseudogene, lncRNA, circRNA, and mRNA can all function as ceRNAs to sponge miRNAs, consequently modulating the de-repression of miRNA targets. Compared with the non-coding RNAs, only a few mRNAs that act as ceRNAs have been mechanically and functionally characterized in the context of AML-associated aberrant gene networks (4-6).

Trdmt1 is also known as Dnmt2, the most conserved member of the DNA methyltransferase family, which has been shown to methylate tRNAs (7). Bone marrow transplantation experiments (8) demonstrated a cell-autonomous defect in hematopoietic stem and progenitor cell differentiation in newborn Trdmt1-deficient mice, suggesting that Trdmt1 is required for cell-autonomous differentiation during hematopoiesis. Although Trdmt1 plays an important role in hematopoiesis, little is known about its action mechanism in AML. miRNA target prediction indicates that Trdmt1 is a putative target gene of miR-181a. Additionally, miR-181a has been reported to be involved in the regulation of myeloid differentiation in AML cells (9,10). Of note, C/EBPα has been validated as a downstream target of miR-181a (11). In our previous study, we have confirmed that a TAT-mediated LIFRα-CT3 prokaryotic expression fusion protein (TAT-CT3) can remarkably induce differentiation in HL-60 cells (12). Based on the above knowledge, we explored the possible ceRNA activity of Trdmt1 mRNA in HL-60 cells and the functional implications of this activity.

## Material and Methods

### Cell culture

The human myeloid leukemia HL-60 cell lines were purchased from the Cell Bank of the Chinese Academy of Sciences (China) and cultured as described previously (13).

### RNA extraction and real-time PCR

Total RNA was isolated from HL-60 cells using Trizol reagent (Takara, China) according to the manufacturer's instructions. For miRNA detection, reverse transcription was performed using miRNA specific stem-loop primers. miR-181a primer, which was purchased from Invitrogen (China), was performed in a real-time PCR detection system. U6 RNA was used as a miRNA internal control. For mRNA detection, the first-strand cDNA was generated using the Reverse Transcription System kit (Takara) with random primers and real-time PCR was performed using a standard SYBR-Green PCR kit protocol in a QuantStudio 6 and 7 Flex Real-Time PCR System (Applied Biosystems, USA). β-actin was used as an endogenous control to normalize the amount of total mRNA in each sample. PCR amplification conditions were 20 s denaturation at 94°C, 20 s annealing at 55°C, and 20 s extension at 72°C for all genes. The oligonucleotide primers are presented in Table 1.

**Table 1:** Sequences of primers used in this study.

Primers	Sequences
Primers for real-time PCR of target genes and miRNAs	
β-actin	
Forward	5′-CTGGCACCACACCTTCTACA-3′
Reverse	5′-AGCACAGCCTGGATAGCAAC-3′
C/EBPα	
Forward	5′-AGACGTCCATCGACATCAGC-3′
Reverse	5′-TTGGCCTTCTCCTGCTGC-3′
Trdmt1-CDS	
Forward	5′-TAGAAGGGACAGGGTCTGTGT-3′
Reverse	5′-TCTTCTCAGGAAATCCGAACTCT-3′
Trdmt1-3′UTR	
Forward	5′-GCTGGTTCCTTACACAAGTCC-3′
Reverse	5′-TCAGATCGTAACAGCTATTCAGC-3
U6	
Forward	5′-CTCGCTTCGGCAGCACATATACT-3′
Reverse	5′-ACGCTTCACGAATTTGCGTGTC-3′
miR-181a	
Forward	5′-CTAGTGAACATTCAACGCTGTC-3′
miR	
Reverse	5′-GTGCAGGGTCCGAGGT-3′
Primers for reverse transcription of miRNAs	
U6 - RT	5′-AAAATATGGAACGCTTCACGAATTTG-3′
miR-181a - RT	5′-GTCGTATCCAGTGCAGGGTCCGAGGT ATTCGCACTGGATACGACACTCACC-3′

### Western blot analysis

Total cell lysates were prepared in 1X SDS buffer. Proteins were separated by SDS-PAGE and transferred to PVDF membranes, which were then blotted with antibodies specific for Trdmt1 (Abcam, UK), C/EBPα (Abcam), and α-tubulin (Sigma, USA). Antigen complexes were visualized using chemiluminescence (Thermo, USA).

### Flow cytometry analysis

HL-60 cells were washed with PBS, resuspended in PBS and incubated with a monoclonal mouse anti-human PE-conjugated anti-CD11b antibody (a granulocytic differentiation marker) for 30 min at 37°C. The fluorescence intensity of stained cells was analyzed by flow cytometry. The results were analyzed by FlowJo software (Treestar, USA), and the positive rate was calculated by subtracting the signals of isotype controls. All the flow cytometry assays were carried out in triplicate.

### Plasmid constructs, oligonucleotides, and cell transfection

For gain-of-function analysis of Trdmt1, coding sequence (CDS) and 3′UTR segments containing the predicted target site of miR-181a of Trdmt1 were amplified by PCR from human cDNA and inserted into pcDNA3.1 and psiCHECK2 luciferase reporter vectors, respectively. For loss-of-function analysis of Trdmt1, siRNA targeting human Trdmt1 and non-targeting negative control were purchased from GenePharma (China). For miR-181a target analysis, site-directed mutagenesis plasmid of the miR-181a target sites in the 3′UTR of Trdmt1 was purchased from Genechem (China). miR-181a mimic and mimic control were purchased from GenePharma and used at a final concentration of 100 nmol/L in transfection following the instructions of the Lipofectamine 3000 kit (Invitrogen). HL-60 cells were transfected with different Trdmt1 constructs at a final concentration of 2.5 μg/mL using the Lipofectamine 3000 kit. For the “rescue” assay, the psiCHECK2-3′UTR constructs or empty vectors at a final concentration of 2.5 μg/mL was co-transfected with the miR-181a mimic or mimic control at a final concentration of 100 nmol/L using the Lipofectamine 3000 kit into HL-60 cells.

### Luciferase reporter assay

HEK-293T cells were co-transfected with psiCHECK2 constructs containing the wild type Trdmt1 or mutant Trdmt1 at a final concentration of 0.2 μg/mL, along with the control vector at a final concentration of 0.2 μg/mL, and miR-181a mimic or mimic control at a final concentration of 0.8 μg/mL, using the Lipofectamine 3000 kit in 24-well plates. The plasmid containing firefly luciferase was used as an internal control. Cells were harvested 48-h post-transfection and assayed with Dual Luciferase Assay (Promega, USA). Data were obtained by normalization of renilla luciferase activity to firefly luciferase activity. All transfection assays were performed in triplicate.

### Statistical analysis

All studies were performed a minimum of three times, and the data are reported as means±SD. The results were considered statistically significant if the P-value was <0.05 as determined by one-way ANOVA or *t*-test.

## Results

### Trdmt1 improved TAT-CT3-induced granulocytic differentiation

**Figure 1:**
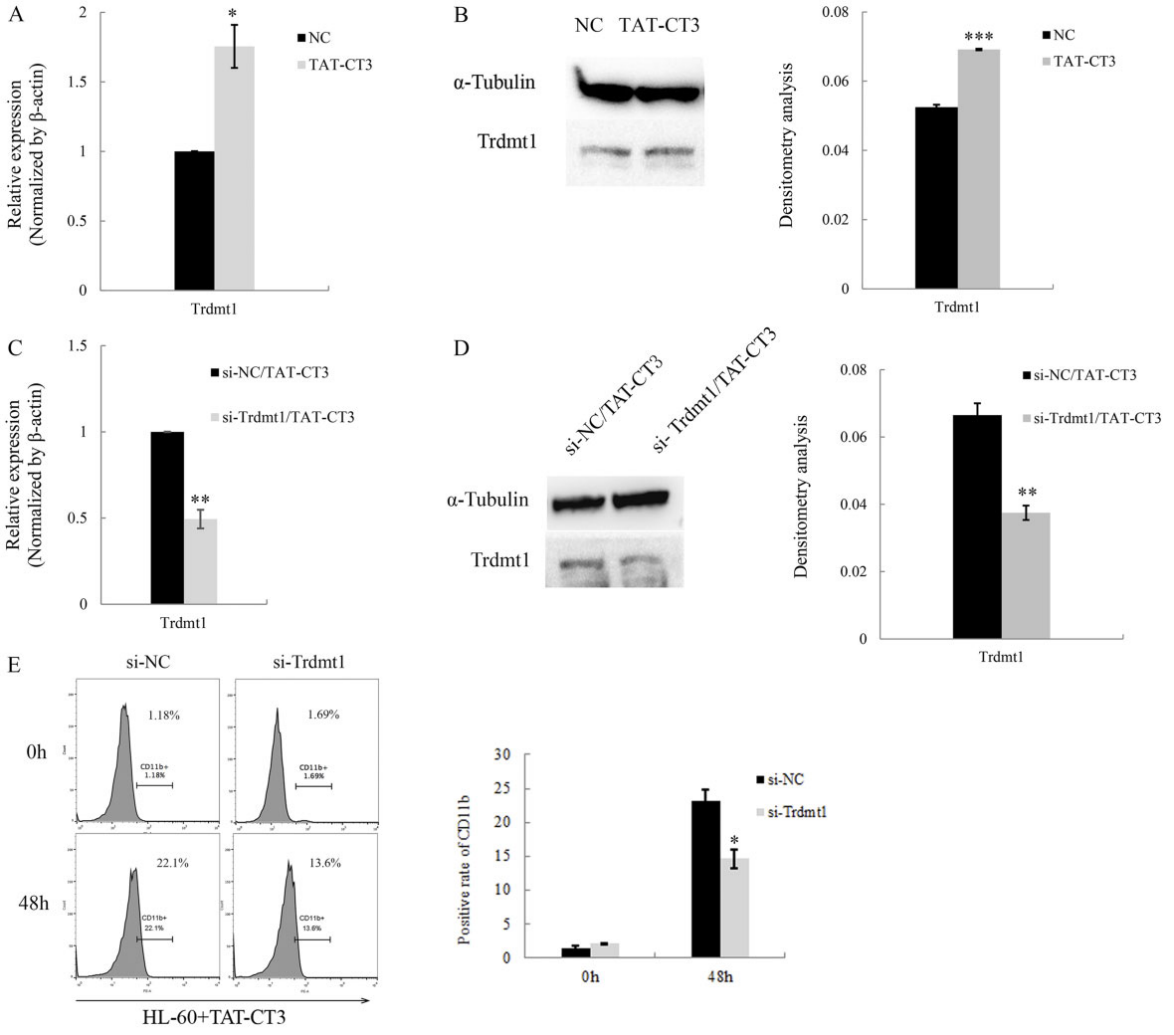
**A**, qPCR analysis of Trdmt1 mRNA in HL-60 cells following 2 days of TAT-CT3 treatment at a concentration of 50 μg/mL. **B**, Western blot analysis and corresponding densitometry analysis of Trdmt1 protein in HL-60 cells treated as described in panel A. **C,** qPCR analysis of Trdmt1 mRNA in HL-60 cells transfected with Trdmt1 siRNAs (si-Trdmt1) and control (si-NC) following 2 days of TAT-CT3 treatment at a concentration of 50 μg/mL. **D,** Western blot analysis and corresponding densitometry analysis of Trdmt1 protein in HL-60 cells transfected with Trdmt1 siRNAs (si-Trdmt1) and control (si-NC) following treatment as described in panel C. **E**, Flow cytometry analysis of the granulocytic differentiation marker CD11b in HL-60 cells transfected with si-Trdmt1 and control, and the percentage of CD11b-positive cells are indicated. Data are reported as means±SD. *P<0.05; **P<0.01; ***P<0.001 (ANOVA or Student's *t*-test).

Knowing that fusion protein TAT-CT3 could induce myeloid differentiation in HL-60 cells (12), we first assayed the expression of Trdmt1 in HL-60 cells after 2 days of treatment with TAT-CT3 to observe changes in the expression of Trdmt1 during TAT-CT3-induced differentiation. Trdmt1 was increased markedly in response to TAT-CT3 treatment compared with the relative control (Figure 1A and B). To further confirm whether Trdmt1 played an important role in HL-60 cell differentiation, siRNA specific to Trdmt1 (si-Trdmt1) was transfected into HL-60 cells and the efficiency of Trdmt1 knockdown was subsequently confirmed by qPCR analysis and western blot (Figure 1C and D). Flow cytometry data demonstrated that Trdmt1 knockdown resulted in lower CD11b+ population than the control group after 2 days of TAT-CT3 treatment (Figure 1E). These results suggested that Trdmt1 played a potential role in myeloid differentiation.

**Figure 2:**
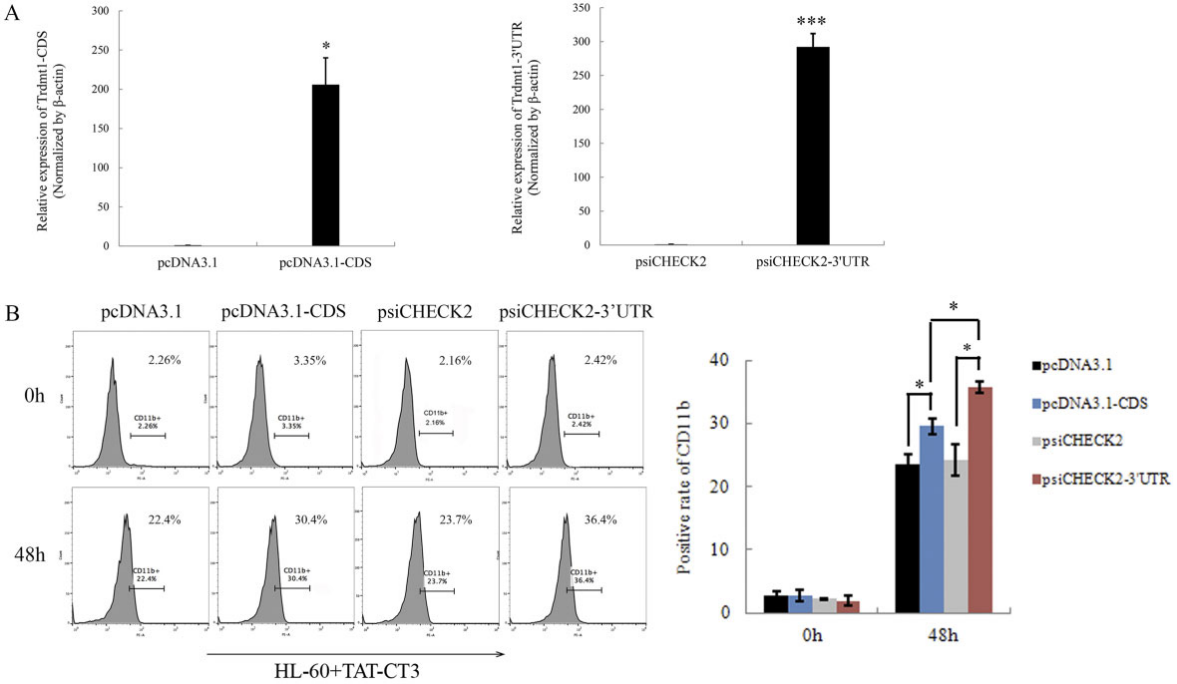
**A,**The expression levels of Trdmt1 3′ untranslated region (UTR) and coding sequence (CDS) in HL-60 cells transfected with a construct expressing Trdmt1 3′UTR (psiCHECK2-3′UTR), Trdmt1 CDS (pcDNA3.1-CDS), and control (psiCHECK2 and pcDNA3.1). **B**, Flow cytometry analysis of CD11b expression in HL-60 cells transfected with a construct expressing Trdmt1 3′UTR (psiCHECK2-3′UTR), Trdmt1 CDS (pcDNA3.1-CDS), and control (psiCHECK2 and pcDNA3.1). Data are reported as means±SD. *P<0.05; ***P<0.001 (ANOVA or Student's *t*-test).

Given the change in the expression of Trdmt1 following TAT-CT3 treatment, we postulated whether Trdmt1 was correlated directly with the induction of HL-60 cell differentiation. We therefore transiently transfected HL-60 cells with Trdmt1 3′UTR, CDS, or with the empty vector as control. After 2 days of transfection, cells were harvested and the expression levels of granulocytic differentiation marker CD11b were detected. The over-expression of both Trdmt1 3′UTR and CDS (Figure 2A) increased the percentage of CD11b+ HL-60 cells after TAT-CT3 treatment compared with the corresponding control groups. In addition, flow cytometry results revealed more CD11b-positive cells in the Trdmt1 3′UTR group (Figure 2B). These data suggested that Trdmt1 3′UTR had a greater impact on myeloid differentiation than Trdmt1 CDS. So we focused our attention on the mechanism of Trdmt1 3′UTR in the subsequent experiments.

### MiR-181a negatively regulated Trdmt1 expression in HL-60 cells

**Figure 3:**
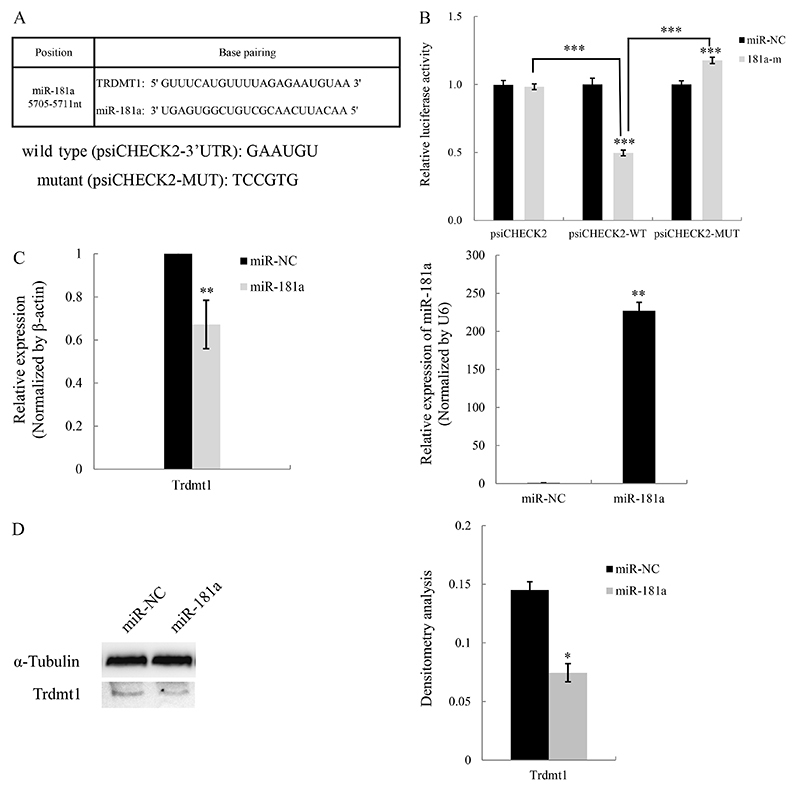
Trdmt1 is a target of miR-181a. **A**, Seed match of Trdmt1 3′ untranslated region (UTR) and hsa-miR-181a. The complementary sequences between Trdmt1 and miR-181a are indicated. **B**, Dual luciferase reporter assay showed that miR-181a mimic reduced relative luciferase activity of Trdmt1-3′UTR but not Trdmt1-MUT (mutant) plasmid. **C**, qPCR analysis of Trdmtm1 mRNA and miR-181a in HL-60 cells transfected with miR-181 mimic and mimic control. **D**, Western blot analysis and corresponding densitometry analysis of Trdmt1 protein in HL-60 cells transfected with miR-181a mimic or mimic control. Data are reported as means±SD. *P<0.05; **P<0.01; ***P<0.001 (ANOVA or Student's *t*-test).

According to the ceRNA theory, mRNAs can regulate one another through their ability to compete for miRNA binding sites (3). To confirm whether Trdmt1 could act as a ceRNA in HL-60 cell differentiation, we searched predicted targets of human miR-181a through miRBase (University of Manchester, UK) and found that the 3′UTR of Trdmt1 gene contained a potential miR-181a binding site (Figure 3A), suggesting its ceRNA potential for miR-181a. Subsequently, we conducted luciferase reporter assays to construct a series of luciferase reporters containing the wild type Trdmt1 including 5705-5711bp positions of Trdmt1 3′UTR (psiCHECK2-3′UTR) or a mutant Trdmt1 (psiCHECK2-MUT), and co-transfected it with control or miR-181a mimic. miR-181a overexpression significantly reduced the luciferase activity of psiCHECK2-3′UTR reporter vector in HEK-293T cells, but had no significant inhibitory effect on psiCHECK2-MUT reporter vector (Figure 3B). Next, we examined whether the endogenous Trdmt1 level was affected by miR-181a. Consistent with the luciferase result, overexpression of miR-181a in HL-60 cells resulted in a significant reduction in Trdmt1 mRNA and protein levels (Figure 3C and D). These data suggested that miR-181a could directly bind to Trdmt1 3′UTR and then repress Trdmt1 in HL-60 cells.

### MiR-181a participated in TAT-CT3-induced granulocytic differentiation by acting as a negative regulator

**Figure 4:**
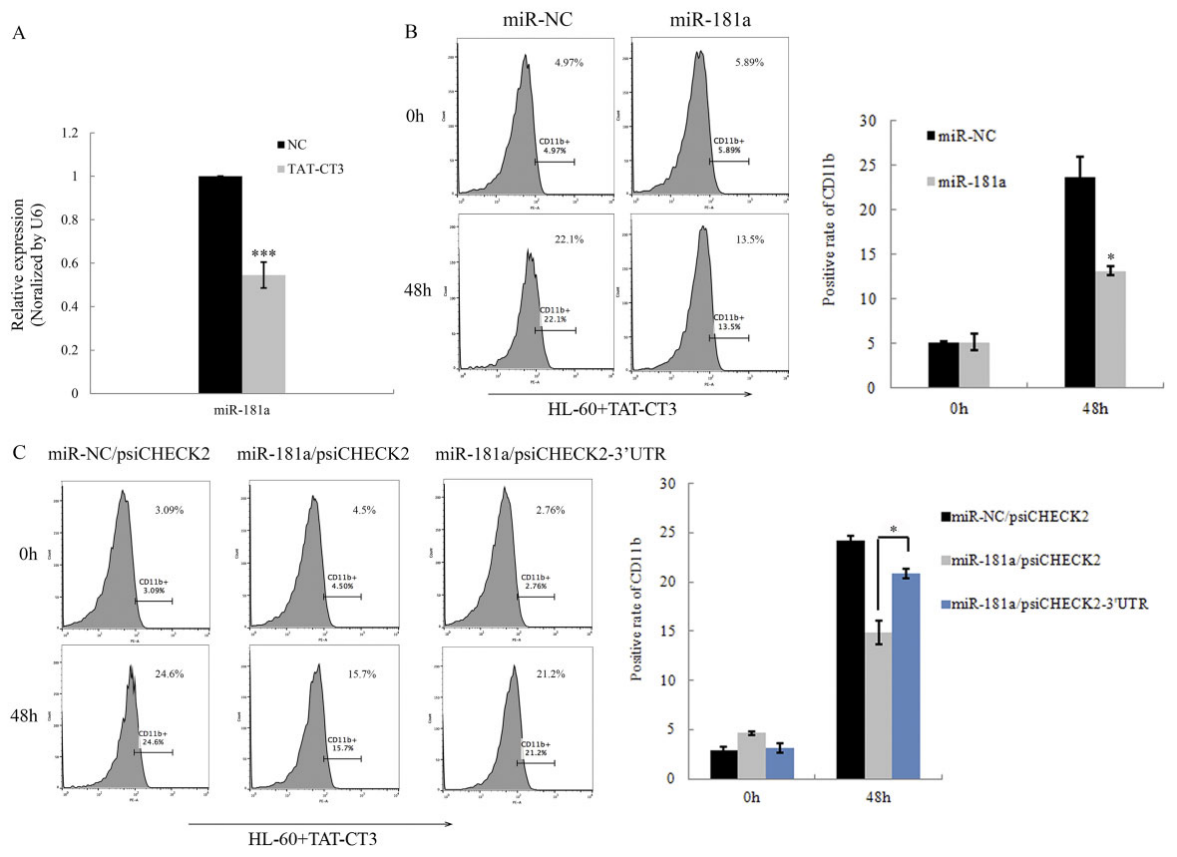
Functional analysis of miR-181a in HL-60 cell differentiation. **A**, qPCR analysis of miR-181a in HL-60 cells following 2 days of exposure to TAT-CT3 at a concentration of 50 μg/mL. **B**, Flow cytometry analysis of CD11b expression in HL-60 cells transfected with miR-181 mimic and mimic control. **C**, Flow cytometry analysis of CD11b expression in the "rescue" assay. Data are reported as means±SD. *P<0.05; ***P<0.001 (ANOVA or Student's *t*-test).

MiR-181a has been reported to be up-regulated in HL-60 cells derived from AML-M2 patients (14,15). We observed changes in miR-181a expression in HL-60 cells with or without TAT-CT3 treatment. qPCR showed that miR-181a was decreased after 2 days of TAT-CT3 treatment compared with the control (Figure 4A), which was opposite to Trdmt1 expression. Then, we analyzed the effect of miR-181a on granulocytic differentiation by transfecting miR-181a mimic into TAT-CT3-treated HL-60 cells. The flow cytometry data revealed a lower percentage of CD11b positive cells in the HL-60 cells transfected with miR-181a mimic (Figure 4B). These data indicated that miR-181a negatively regulated TAT-CT3-induced granulocytic differentiation.

To further determine whether the functional relevance of miR-181a in AML was regulated by Trdmt1 ceRNA activity, we performed a “rescue” assay by co-transfecting psiCHECK2-3′UTR and miR-181a mimic into HL-60 cells following TAT-CT3 treatment, and found that reintroduction of Trdmt1 3′UTR could rescue the miR-181a-mediated blockage on cell differentiation (Figure 4C). These data indicated that Trdmt1 could function as a ceRNA in leukemia cell differentiation.

### ceRNA activity of Trdmt1 3′UTR affected C/EBP&mac_agr; in HL-60 cells

**Figure 5:**
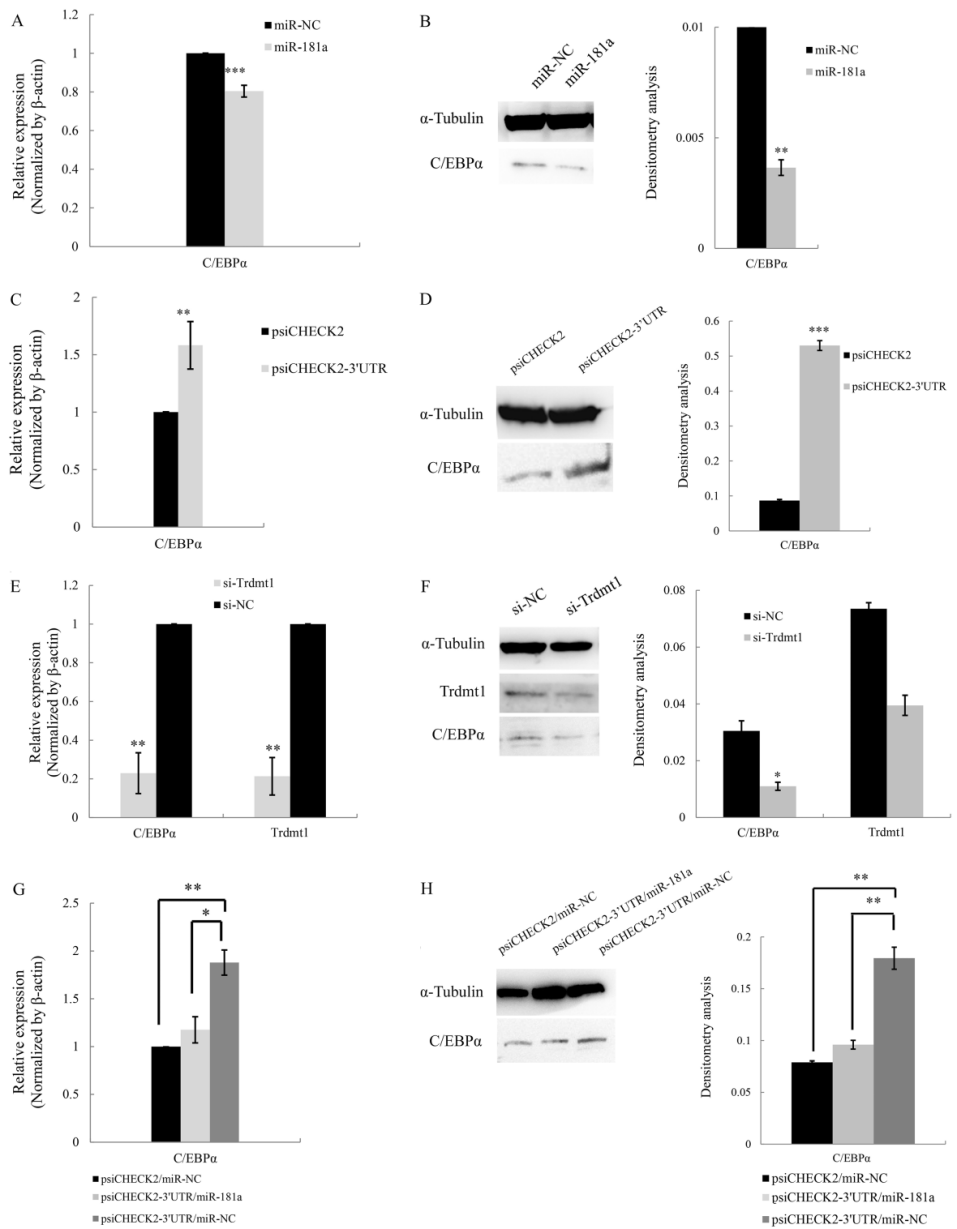
Regulation of C/EBPα by Trdmt. **A**, qPCR analysis of C/EBPα mRNA in HL-60 cells transfected with miR-181 mimic and mimic control. **B**, Western blot analysis and corresponding densitometry analysis of C/EBPα protein in HL-60 cells transfected with miR-181a mimic or mimic control. **C**, qPCR analysis of C/EBPα in HL-60 cells transfected with Trdmt1 3′ untranslated region (UTR) (psiCHECK2-3′UTR) and control (psiCHECK2). **D**, Western blot analysis and corresponding densitometry analysis of C/EBPα protein in HL-60 cells treated as described in panel C. **E**, qPCR analysis of C/EBPα and Trdmt1 mRNA in HL-60 cells transfected with Trdmt1 siRNAs (si-Trdmt1) and control (si-control). **F**, Western blot analysis and corresponding densitometry analysis of C/EBPα and Trdmt1 protein in HL-60 cells treated as described in panel E. **G**, qPCR analysis of C/EBPα mRNA in HL-60 cells transfected with control, Trdmt1-3′UTR, or Trdmt1-3′UTR plus miR-181a mimic. **H**, Western blot analysis and corresponding densitometry analysis of C/EBPα protein in HL-60 cells treated as described in panel G. Data are reported as means±SD. *P<0.05; **P<0.01; ***P<0.001 (ANOVA or Student's *t*-test).

C/EBPα is a potential target of miR-181a, a validated gene for granulocytic maturation. MiR-181a can directly regulate C/EBPα expression in macrophages (11). To identify whether miR-181a could also target C/EBPα in AML cells, we investigated the expression of C/EBPα in HL-60 cells. qRT-PCR analysis and western blot analysis showed that both C/EBPα mRNA and protein levels were decreased in HL-60 cells transfected with miR-181a mimic compared with the control (Figure 5A and B).

**Figure 6:**
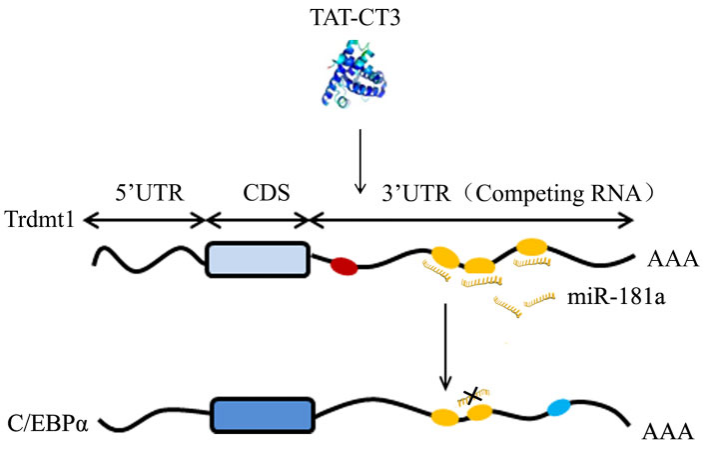
Illustration showing the mechanism of Trdmt on ceRNA. CDS: coding sequence; UTR: untranslated region; ceRNA: competing endogenous RNA.

To determine whether Trdmt1 could act as a ceRNA to affect C/EBPα, we first detected the mRNA and protein levels of C/EBPα. As expected, overexpression of Trdmt1 3′UTR increased both mRNA and protein levels of C/EBPα (Figure 5C and D), while Trdmt1 knockdown decreased the expression of C/EBPα (Figure 5E and F). In addition, transfection of Trdmt1 3′UTR alone or together with miR-181a mimic into HL-60 cells was performed to assay ceRNA activity of Trdmt1. As shown in Figure 5G and H, miR-181a restoration upon psiCHECK2-3′UTR abrogated this increase. The above data confirmed the hypothesis that Trdmt1 modulated C/EBPα by competitively recruiting endogenous miR-181a in HL-60 cells. The illustration for the mechanism of Trdmt1 on ceRNA is summarized in Figure 6.

## Discussion

The prevalent view is that protein-coding genes must be translated into a protein to exert function. Recent studies indicated exogenous expression of the 3′UTR constructs such as uPAR, RUNX1T1, and C-Myc participate in leukemia cells migration and differentiation by regulating gene expression ([Bibr B04] [Bibr B05] 46). As the most conserved member of the DNA methyltransferase family (7), Trdmt1 is found to be crucial for differentiation both in the hematopoietic system and bone marrow mesenchymal stem cells (8). In the present study, our data showed that overexpression of Trdmt1 3′UTR promoted TAT-CT3-induced granulocytic differentiation in HL-60 cells. It provided evidence for the hypothesis that protein-coding genes play roles through the 3′UTR of their mRNA in leukemia.

ceRNAs are implicated in many biological processes. Disruption of the equilibrium between ceRNAs and miRNAs is critical for tumorigenesis. As a transcript encoding gene, most mRNAs have binding regions to the seed sequences of some miRNAs, especially at the 3′UTR (16). Therefore, 3′UTRs are the essential elements of the ceRNA cross-talk. Ample evidence has shown that non-coding RNAs such as lncRNAs and circRNAs can function as ceRNAs to modulate proliferation, apoptosis, differentiation, and chemoresistance of AML cells through competing for the binding of miRNAs (17-24). Here, we used ceRNA hypothesis to explain the regulatory function of Trdmt1 3′UTR based on our experimental results. We found that Trdmt1 3′UTR acts in trans to modulate C/EBPα levels and it can function as a ceRNA of C/EBPα by competitively binding to miR-181a in HL-60 cells. C/EBPα is a transcription factor specific for myeloid cell differentiation. It plays a key role in the differentiation and maturation of myeloid cells (25). Our research proved that Trdmt1 3′UTR promoted granulocytic differentiation of HL-60 cells by up-regulating the expression of C/EBPα.

In our current study, we constructed Trdmt1 3′UTR segments that only contained miR-181a binding site. Furthermore, bioinformatics analysis of the Trdmt1 3′UTR has shown that miR-101 and miR-26 can also interact with Trdmt1 3′UTR. Some studies have reported that miR-101 and miR-26 are considered to be a tumor suppressor in leukemia (26,27). We suppose the long sequence of the 3′UTR containing these miRNAs binding sites may exert complex biological functions. We predict the effectiveness of 3′UTR will depend on the number of miRNAs that it can sponge.

Moreover, of particular note, mRNA exhibited effects on gene expression via protein-coding function in addition to its ceRNA activity. It is known that protein and mRNA derived from an identical gene may exert the same or different biological effects. Hmga2 promoted lung carcinogenesis both as a protein-coding gene and as a ceRNA dependent upon the presence of let-7 sites (28,29). ZEB2 protein was recognized as an activator of epithelial-mesenchymal transition (EMT) (30). Later study validated ZEB2 mRNA as a bonafide ceRNA for PTEN (31). Herein, we confirmed that Trdmt1-coded protein has an effect on HL-60 cells differentiation as well, suggesting that Trdmt1 might have a role of pro-differentiation in some leukemia cells. These discoveries may change the way we look at the coding transcriptome.

In summary, this study provided evidence for the first time that Trdmt1 3′UTR acted as a natural sponge to bind miR-181a and inhibit its function, which may help gain a better understanding about the molecular mechanism underlying AML cell differentiation. The Trdmt1/miR-181a/C/EBPα axis may provide a clue for better therapeutic strategy in the future treatment of AML.
